# Supplementary material for: Prediction and interpretation of deleterious coding variants in terms of protein structural stability
Source: Sci Rep. 2018 Mar 14;8:4480. doi: 10.1038/s41598-018-22531-2 (PMC5852127; doi:10.1038/s41598-018-22531-2)
Supplement: Supplementary file 1 — Supplementary Information [file 41598_2018_22531_MOESM1_ESM.pdf]

Supporting Information
Prediction and interpretation of deleterious coding variants in terms of protein structural stability

François Ancien, Fabrizio Pucci, Maxime Godfroid, Marianne Rooman
Department of BioModeling, BioInformatics & BioProcesses, Université Libre de Bruxelles and Interuniversity Institute of Bioinformatics in Brussels, Belgium

Non-synonymous SNV training set

The SNV training set **S** is built as described in the first Methods subsection in the main text. The list of entries is given in Table S1 (additional tds file).

Non-synonymous SNV test sets

We considered four test sets, obtained by refining the datasets used by VIPUR.[1] They are given in Tables S2-S5 (additional tds files):

- $\mathbf{S_H}$: The Human variant dataset (Table S2) is composed of 1,252 variants inserted in 573 human proteins with experimental or modeled structures; the latter were obtained through comparative modeling based on a template of 30% sequence identity at least. We filtered out the mutations from our training dataset **S** and from HumDiv[2] (on which Polyphen-2 is trained).

- $\mathbf{S_{NH}}$: The Non-Human variant dataset (Table S3) contains 2,922 variants in 1,233 non-human proteins with experimental or modeled structures obtained through comparative modeling based on a template of 30% sequence identity at least. This set is part of the training set of the VIPUR predictor,[1] but there is no overlap with the training sets of the other predictors, which contain only human variants.

- $\mathbf{S_{CV}}$: The ClinVar dataset (Table S4) includes 2,510 variants inserted in 407 proteins with experimental or modeled structures obtained in,[1] and that have been annotated in the original ClinVar dataset[3] as pathogenic, likely pathogenic, benign or likely benign. This set has some overlap with the training sets of the tested predictors. No information is available about the sequence identity between the target sequences and template structures used for comparative modeling.

- $\mathbf{S_{SSC}}$: The Simon Simplex dataset (Table S5) is a collection of 2180 *de novo* missense variants originally gathered from the Simons Simplex Collection (SSC) of sequenced exomes from families with children affected by Autism Spectrum Disorders (ASD) and unaffected siblings.[4] They have been mapped to their experimental or modeled protein structures in[1] and the mapping has been further checked in this investigation. Note that due to the complexity of the ASDs, some of these variants in probands may be non-causal for ASD or contribute only weakly to the phenotypes. The overlap between this dataset and the training sets of the different predictors is limited. No information is available about the sequence identity between the target sequences and their template structures used for comparative modeling.

Statistical potentials

To estimate the deleteriousness of mutations, we used several types of statistical potentials as well as volume terms and solvent accessibility (see Eqs (1-2) in the main text). They are listed in Table S6. They can be grouped into three basis types: (1) distance potentials describing tertiary interactions, in which one of the structure descriptors is the distance d between the average side chain geometric centers of two amino acids s; (2) solvent accessibility potentials, in which one of the structure descriptors is the solvent accessibility a of a given residue and s the type of an amino acid situated in a sequence window around this residue; (3) torsion potentials describing local interactions along the sequence, in which one of the structure descriptors is the main chain torsion angle domain t of a residue and s the type of amino acid in the sequence neighborhood. Several combinations of these basic potentials were used.

The subset of the potentials listed in Table S6, which are used in our different predictors, are:

- PoPMuSiC (Eq. 3 in the main text): all 13 potentials.

- HoTMuSiC (Eq. 6): the first 9 potentials.

- ANN (Eq. 8) and SNPMuSiC (Eq. 11): the first 11 potentials.

- PNN (Eq. 9): 6 combinations of potentials, in order to reduce the computational times. These are $\Delta W_1 = \Delta W_{sa} + \Delta W_{ssa}$, $\Delta W_2 = \Delta W_{sd}$, $\Delta W_3 = \Delta W_{sds}$, $\Delta W_4 = \Delta W_{sad} + \Delta W_{sadsa}$, $\Delta W_5 = \Delta W_{st} + \Delta W_{stt}$ and $\Delta W_6 = \Delta W_{std} + \Delta W_{stdst}$.

N	Potential or feature	Type	KS-test P-value
1	ΔW_{st}	torsion	$< 10^{-5}$
2	ΔW_{stt}	torsion	< 0.1
3	ΔW_{sst}	torsion	< 0.005
4	ΔW_{sa}	accessibility	$< 10^{-7}$
5	ΔW_{saa}	accessibility	< 0.0005
6	ΔW_{sd}	distance	$< 10^{-10}$
7	ΔW_{sds}	distance	$< 10^{-10}$
8	ΔW_{std}	distance	< 0.0001
9	ΔW_{sad}	distance	$< 10^{-10}$
10	ΔW_{ssa}	accessibility	< 0.005
11	ΔW_{sta}	torsion + accessibility	< 0.15
12	ΔW_{stdst}	torsion + distance	< 0.0001
13	ΔW_{sadsa}	accessibility + distance	< 0.00001
14	ΔV	volume	$< 10^{-10}$
15	A	accessibility	$< 10^{-10}$

Table S6. Potentials and structural features utilized in this investigation. The subscripts of the potentials ΔW refer to the type of sequence and structural elements from which they are built, using Eqs (1-2) in the main text. The interresidue distances d range from 3.0 to 8.0 Å and are grouped into 25 bins of 0.2 Å width.[5] The residue solvent accessibilities a are defined as the ratio between the solvent accessible surface in the given structure and in an extended tripeptide Gly-X-Gly conformation;[6] they are discretized into five bins. The main chain torsion angles are divided into seven domains t.[7] The last column contains the P-value of the Kolmogorov-Smirnov (KS) test that compares the distributions of the change in folding free energy, in accessibility and in volume for deleterious and neutral SNVs.

Performance of our predictors in cross validation on the training set

In the main text, we computed the performance of our predictors in 5-fold cross validation at the mutation level, which consists in training the model on 4/5 randomly chosen mutations that belong to the **S** training set and applying it to the remaining entries. The results are shown in Table 1 of the main text. Here we applied a different 5-fold cross validation procedure, at the protein level rather than on the mutation level, in which the proteins rather than the mutations are divided into five subsets. The results are shown in Table S7. No significant differences are observed between the two types of cross-validation scores.

Method	Sensitivity	Specificity	PPV	NPV	BACC	AUROC
PoPMuSiC	0.62	0.64	0.84	0.36	0.63	0.68
HoTMuSiC	0.58	0.66	0.84	0.34	0.62	0.66
Solvent Accessibility	0.71	0.66	0.86	0.42	0.68	0.72
PNN	0.72	0.70	0.88	0.44	0.71	0.76
ANN	0.70	0.73	0.89	0.55	0.71	0.76
PROVEAN	**0.85**	0.58	0.86	**0.56**	0.72	0.80
SNPMuSiC	0.77	**0.73**	**0.90**	0.51	**0.75**	**0.82**

Table S7. Performance of the different prediction methods in protein-based 5-fold cross validation on the learning set. Sensitivity is defined as $TP/(TP+FN)$, specificity as $TN/(TN+FP)$, positive predictive value (PPV) as $TP/(TP+FP)$, and negative predictive value (NPV) as $TN/(TN+FN)$. BACC is the average between sensitivity and specificity, and AUROC is the Area Under the Receiver Operating Characteristic curve. The scores are averages on the 5-fold cross-validation experiments. The values in bold indicate the highest scores in each category; the AUROC score in bold is statistically different from the other AUROC scores, as estimated by DeLong's test.

Performance comparisons on the test sets

We compared the performance of our predictors with that of commonly used predictors that do not utilize contextual information, *i.e.* Polyphen-2,[2] Provean,[8,9] CADD,[10] SIFT[11] and VIPUR,[1] on the four test sets $\mathbf{S_H}$, $\mathbf{S_{NH}}$, $\mathbf{S_{CV}}$, and $\mathbf{S_{SSC}}$ described in Tables S2-S5. The results of these programs are taken from[1] when available. The performance comparison on the sets $\mathbf{S_H}$ and $\mathbf{S_{NH}}$ is described in the main text (Table 2). Here we show the performances on the two other sets $\mathbf{S_{CV}}$ and $\mathbf{S_{SSC}}$, which contain neutral and disease-causing human variants from ClinVar[3] and *de novo* missense mutations of the Simon Simplex Collection (SSC),[4] respectively. The protein structures in these sets are obtained by comparative modeling. However, as the sequence identity between the target and template sequences is not available (unlike those for the sets $\mathbf{S_H}$ and $\mathbf{S_{NH}}$), we could not set a threshold value of 30% on this sequence identity to filter out the incorrect 3D models. Also, we could not set a limit on the X-ray resolution of the template protein structures. The fact that some of the structural models are probably of poor quality explains why sequence-based predictors sometimes perform better on the $\mathbf{S_{CV}}$ set. In spite of this, SNPMuSiC has the highest PPV score and specificity on both sets.

Method	Sensitivity	Specificity	PPV	NPV	BACC	AUROC
**ClinVar**						
VIPUR	0.75	0.76	0.90	0.51	0.75	0.84
Polyphen-2	**0.88**	0.63	0.87	0.65	0.76	0.86
SIFT	0.81	0.69	0.88	0.56	0.75	0.82
CADD	0.87	0.74	0.90	**0.67**	**0.80**	**0.87**
Provean	0.81	0.79	0.92	0.60	**0.80**	**0.87**
SNPMuSiC	0.65	**0.87**	**0.93**	0.47	0.76	0.82
**Simon Simplex Collection**						
SIFT	0.50	0.55	0.64	0.41	0.53	**0.53**
Polyphen-2	0.58	0.46	0.63	0.41	0.52	**0.53**
VIPUR	0.41	0.63	0.64	0.41	0.52	**0.53**
BLOSSUM62	0.49	0.59	0.65	**0.42**	**0.54**	**0.54**
CADD	**0.59**	0.41	0.61	0.39	0.50	0.50
Provean	0.53	0.54	0.64	**0.42**	0.53	**0.54**
SNPMuSiC	0.36	**0.72**	**0.67**	**0.42**	**0.54**	**0.54**

Table S8. Comparison of the performances of different predictors on the test sets $\mathbf{S_{CV}}$ and $\mathbf{S_{SSC}}$. Note that the training sets of the different predictors have some limited overlap with these two test sets. The values in bold indicate the highest scores in each category; the AUROC scores that are not significantly different from the highest score (as estimated by a DeLong-test P-value ≥ 0.05) are also in bold.

References

1. Baugh, E. H., Simmons-Edler, R., Müller, C.L., Alford, R.F., Volfovsky, N., Lash, A.E., Bonneau, R. Robust classification of protein variation using structural modelling and large-scale data integration. Nucleic Acids Res. 44, 2501-2513 (2016).

2. Adzhubei, I. A., Schmidt, S., Peshkin, L., Ramensky, V.E., Gerasimova, A., Bork, P., Kondrashov, A.S., Sunyaev, S.R. A method and server for predicting damaging missense mutations. Nat. Methods 7, 248-249 (2010).

3. Landrum, M.J., Lee, J.M., Riley, G.R., Jang, W., Rubinstein, W.S., Church, D.M., Maglott, D.R. ClinVar: public archive of relationships among sequence variation and human phenotype, Nucleic Acids Res 42, D980-5 (2014).

4. O'Roak, B.J., Vives, L., Girirajan, S., Karakoc, E., Krumm, N., Coe, B.P., Levy, R., Ko, A., Lee, C., Smith, J.D., Turner, E.H., Stanaway, I.B., Vernot, B., Malig, M., Baker, C., Reilly, B., Akey, J.M., Borenstein, E., Rieder, M.J., Nickerson, D.A., Bernier, R., Shendure, J., Eichler, E.E. Sporadic autism exomes reveal a highly interconnected protein network of de novo mutations, Nature 485, 246-250 (2012).

5. Kocher, J. P., Rooman, M. J., Wodak, S. J. Factors influencing the ability of knowledge-based potentials to identify native sequence-structure matches. J. Mol. Biol. 235, 1598-1613 (1994).

6. Dehouck, Y., Gilis, D., Rooman, M. A new generation of statistical potentials for proteins. Biophys. J. 90, 4010-4017 (2006).

7. Rooman, M., Kocher, J.P., Wodak, S. Prediction of protein backbone conformation based on seven structure assignments: Influence of local interactions. J. Mol. Biol. 221, 961-979 (1991).

8. Choi, Y., Sims, G. E., Murphy, S., Miller, J. R., Chan, A. P. Predicting the Functional Effect of Amino Acid Substitutions and Indels. PLoS One 7, e46688 (2012).

9. Choi, Y. and Chan, A. P. PROVEAN web server: a tool to predict the functional effect of amino acid substitutions and indels. Bioinformatics 31, 2745-2747 (2015).

10. Kircher, M., Witten, D.M., Jain, P., O'Roak, B.J., Cooper, G.M., Shendure, J. A general framework for estimating the relative pathogenicity of human genetic variants. Nat. Genet. 46, 310-315 (2014).

11. Kumar, P., Henikoff, S., Ng, P. C. Predicting the effects of coding non-synonymous variants on protein function using the SIFT algorithm. Nat. Protoc. 4, 1073-1081 (2009).
